# A New Challenge for the Old Excipient Calcium Carbonate: To Improve the Dissolution Rate of Poorly Soluble Drugs

**DOI:** 10.3390/pharmaceutics15010300

**Published:** 2023-01-16

**Authors:** Valeria Ambrogi

**Affiliations:** Department of Pharmaceutical Sciences, Via del Liceo 1, 06123 Perugia, Italy; valeria.ambrogi@unipg.it; Tel.: +39-075-5855125

**Keywords:** calcium carbonate, porous calcium carbonate, functionalized calcium carbonate, poorly soluble drug, solid dispersion, dissolution rate, physical stability

## Abstract

Calcium carbonate is an excipient traditionally used in solid dosage forms with several functions such as a diluent, a quick dissolution agent, a buffer and an opacifier. Recently, many other challenges have arisen for calcium carbonate and, among them, the possibility of using it as an excipient for improving the dissolution rate of poorly soluble drugs. As a consequence of their poor solubility in biological fluids, many active ingredients suffer from low and erratic bioavailability when administered by the oral route and thus, many formulation strategies and excipients have been proposed to overcome this problem. Among them, calcium carbonate has been proposed as an excipient for improving dissolution rates. Calcium carbonate has many interesting characteristics, in fact it dissolves quickly in gastric fluid, is inexpensive and is safe. It exists in different polymorphic forms and in porous morphology and recently a porous functionalized calcium carbonate has been proposed as a new excipient. This review is the first overview on the use of calcium carbonate as an excipient for improving drug dissolution rates. The drug loading procedure, the physical characterization of the drug/CaCO_3_ samples and their dissolution profiles will be described. Moreover, the possible mechanisms of dissolution improvement, such as the presence of the drug in amorphous or polymorphic forms, in small crystals, and the effects of CaCO_3_ dissolution in acidic medium will be discussed. Different polymorphic forms of calcium carbonate and the presence of porosity and functionalization will be analyzed as well and their effects on dissolution rates will be discussed.

## 1. Introduction

Drug solubility has a crucial role for the drug oral administration route. This is proven by the Biopharmaceutic Classification System (BCS) which classifies drugs for oral administration into four different classes based on their aqueous solubility/dissolution and permeability [1]. Drugs with: (i) high permeability and high solubility belong to class 1, (ii) high permeability but low solubility to class 2, (iii) low permeability and high solubility to class 3 and iv) both low solubility and low permeability to class 4.

**Figure 1:**
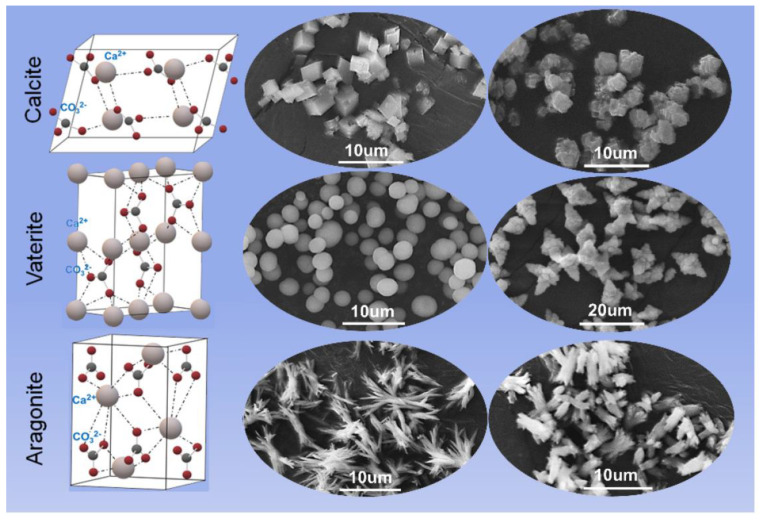
Crystal structure and morphology of three polymorphs of CaCO_3_, reproduced from [18], 2022, MDPI.

Drugs owing to class 2 and 4 have often low and variable bioavailability because of their low solubility in physiological fluids. In fact, generally, a compound whose aqueous solubility is <100 μg/mL is considered to have limited bioavailability and the limiting step of its absorption is its dissolution rate [2]. Moreover, poor water solubility affects numerous stages during the drug development as the need for solubilizing the drug manifests itself right from the beginning of the investigation and the preclinical studies. Among newly discovered agents with pharmaceutical activities, about 90% are characterized by poor water solubility and about the 40% of the commercial medicines are formulated with poorly soluble drugs [3]. Thus, with of the aim of improving drug bioavailability and allowing new pharmaceutical entities to reach the market, many technological strategies have been investigated for improving dissolution rates and for solving problems related to poor solubility. Chemical techniques that have been employed are the formation of prodrugs and the use of different salts [3,4,5]. The main physical strategies are micronization, the use of polymorphic or amorphous forms and co-crystals, and the formulation strategies include solid dispersions, inclusion complexes and solid-liquid techniques [3,4,5,6,7,8,9]. The presence of excipients such as surfactants, polymers, super-disintegrants and multifunctional excipients can also accelerate drug release [10,11]. Excipients can act with different mechanisms such as forming inclusion complexes with drug molecules, promoting faster and/or more extensive disgregation, changing the pH of the microenvironment, improving drug crystal wetting and can affect the formation and stabilization of suitable polymorphic or amorphous drug forms [10,11]. Thus, excipients can have a key role in increasing the dissolution rate/apparent solubility of drugs and, recently, among them, calcium carbonate (CaCO_3_) has been investigated because of its many positive characteristics. CaCO_3_ is a safe excipient traditionally used as a diluent for directly compressible tablets [10]. When in contact with gastric acid, it reacts to form soluble calcium chloride and carbon dioxide. CaCO_3_ is used also for its buffering properties, as a superdisgregant agent in dispersible/effervescent forms and as a bulking agent in tablet sugar-coating and as an opacifier in film-coating for tablets [12,13,14]. CaCO_3_ has three main polymorphic forms (Figure 1): calcite, the most thermodynamically stable phase, vaterite and aragonite [15]. Calcite and aragonite are the two most frequent phases present in nature. When in an amorphous form, CaCO_3_ is highly unstable and rapidly transforms to vaterite and successively to calcite. Vaterite is metastable and transforms to either aragonite or calcite upon contact with water. Calcite crystals are rhombohedral, aragonite has needle crystals and mainly exists in the shell of mollusks, whereas vaterite forms cauliflower-like spheres or disks which are built by smaller entities [16,17,18]. Thus, the vaterite crystals are obtained by assembly into larger particulate structures and this makes the vaterite phase porous with a relatively high surface area in comparison with calcite and aragonite.

Calcite has numerous industrial applications such as filler in paper, plastics, rubber, paints and foodstuffs, due to its high stability and mechanical strength [19,20]. Aragonite has been proposed as a scaffold for bone repair and tissue engineering and other biomedical applications [21] whereas vaterite, due to both its porosity and surface area, has good properties for application in drug delivery [22]. In fact, the porous morphology and developed internal structure allow it to host both macromolecules and low molecular weight compounds which can be loaded by co-precipitation during vaterite formation or by adsorption [22].

Recently, CaCO_3_ has been proposed as an excipient with numerous innovative functions, for example as a carrier for obtaining pH-responsive drug delivery systems for cancer treatments [23], as a versatile material for the controlled delivery of antimicrobials [24] and other drugs and as a gene delivery nanocarrier [25,26]. Emerging interest is devoted also to CaCO_3_ capability of improving the dissolution rate of poorly water-soluble drugs.

This review focuses its attention on the use of CaCO_3_ as an excipient to enhance drug dissolution. Drug loading procedures, the chemical–physical characteristics of drug/CaCO_3_, and in vitro drug release studies have been described to identify the parameters that can affect the drug solubility and release profile. Finally, attention has been devoted to the porous and mesoporous CaCO_3_ as pores improve the particle specific surface area with increase in the adsorption capability and the physical stability of the loaded drug.

## 2. CaCO_3_ as an Excipient for Dissolution Improvement of Poorly Water-Soluble Drugs

**Figure 2:**
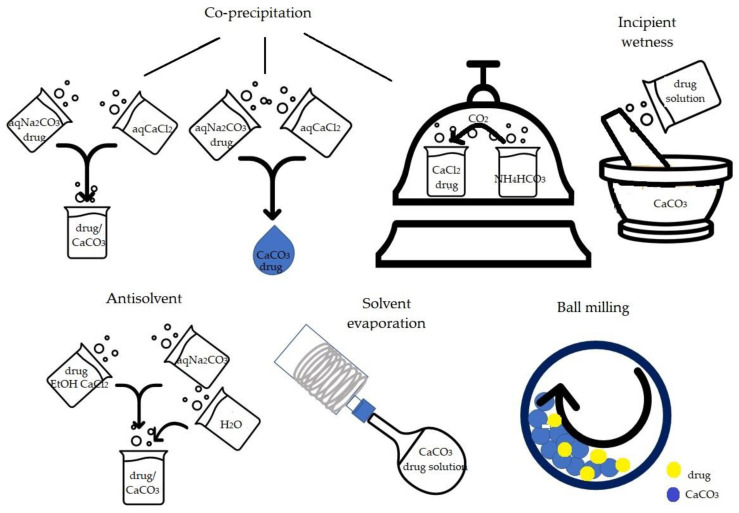
Schematic representation of the main procedures to obtain drug-carbonate samples.

The main procedures described for the preparation of CaCO_3_/drug samples are ball milling, co-precipitation, solvent evaporation, antisolvent method and incipient wetness, also called wet impregnation (Figure 2). Ball milling consists in milling CaCO_3_ and drug powders for a determined time and frequency. The co-precipitation procedure consists of inducing the formation and precipitation of CaCO_3_ in the presence of the drug, which is incorporated in CaCO_3_ crystals during CaCO_3_ nucleation and precipitation. The solvent evaporation process involves the drug dissolution in organic solvent, the successive addition of CaCO_3_ and finally solvent evaporation. The antisolvent procedure consists of the co-precipitation induced by mixing an ethanolic CaCl_2_ and drug solution with a Na_2_CO_3_ aqueous solution in which both drug and the forming CaCO_3_ are insoluble and successively dilution with water which acts as an antisolvent. Lastly, the incipient wetness, used for functionalized calcium carbonate (FCC), consists of mixing a concentrated drug solution with CaCO_3_ to obtain a wet powder which is finally dried.

Table 1 collects different CaCO_3_ forms and drugs and main methods for obtaining carbonate-drug composites.

### 2.1. Ball Milling

The first paper which describes the effect of CaCO_3_ on the solubilization of poorly soluble drugs was published in 2005 [27]. In this paper the effects of the presence of insoluble additives such as CaCO_3_ on drug solubilization was studied. Several physical mixtures (2:1 drug-calcite, by mole) of sulfathiazole and CaCO_3_, characterized by different particles sizes, were prepared by ball milling. Then, the mixtures were submitted to dissolution at 37 °C in deionized water pH 6. The addition of calcite to sulfathiazole improves the dissolution rate of both components with an increase in the amount of the dissolved drug. This effect was observed, even if in the lower extent, but also in the case of the simple physical mixture and it was more evident for the mixture composed of sulfathiazole particles larger than those of CaCO_3_. After mechanical treatment a further increase in the solubility was observed, and the Authors explain this effect with the improvement of the contact between the two components with the formation of a mechano-composite sulfathiazole-CaCO_3_. No physical–chemical characterizations such as X-ray diffraction and thermal analysis have been performed to confirm the hypothesis.

### 2.2. Co-Precipitation

Maver et al. obtained naproxen/CaCO_3_ composites by co-precipitation [28] by mixing an aqueous solution of CaCl_2_^.^6H_2_O with a solution of naproxen and Na_2_CO_3_. Two samples were prepared by simple mixing the two solutions and changing the way of drying (heating at 50 °C for one sample and freeze-drying for the other). A third sample was obtained changing the mixing procedure. In fact, in this case the solutions were added from two needles with tips so close that a unique droplet was obtained. It was immediately filtered and dried by heating. All samples showed a low drug loading (3.4%), but a different surface area, which was higher for the freeze-dried sample (14.24 m^2^/g vs. 3.23 m^2^/g and 3.87 m^2^/g for other samples). In all samples, crystalline naproxen was detected by X-ray diffraction and differential thermal analysis. Moreover, the sample obtained by simple mixture and drying at 50 °C revealed the presence of CaCO_3_ as calcite, whereas the other two samples showed the presence of vaterite. A dissolution test, performed in acid fluid at pH 1.2, exhibited an improved drug release from all the samples in comparison with the pure naproxen. Samples containing vaterite showed a burst effect within the first 20–30 min. which was less evident for the sample containing calcite. Successively, the drug was released slowly from all the samples and after 300 min it was not completely released. The release was attributed to the rapid but partial solubilization of CaCO_3_. The dissolution is higher for vaterite in comparison with calcite and this explains the higher burst effect for the two samples containing CaCO_3_ in the form of vaterite. The Authors highlight the possibility of preparing composites with a suitable drug release based on the CaCO_3_ polymorphic form. This study posits that the vaterite polymorph has a better effect than the calcite form on drug dissolution. Unfortunately, this procedure allows for a very low drug loading.

The co-precipitation procedure was also proposed by Zhou for curcumin/CaCO_3_ [29]. In this case the co-precipitation was induced via CO_2_ diffusion into an ethanolic solution of curcumin and CaCl_2_. Samples with increasing curcumin amount were prepared and, for comparison, a composite, obtained by adding NH_4_HCO_3_ to an ethanolic solution of CaCl_2_ and curcumin, was prepared. The co-precipitation under CO_2_ diffusion gave rise to better drug entrapment and samples with an acceptable drug content were obtained (42.6% *w*/*w* vs. 29.9% *w*/*w* for the control). Characterization revealed that curcumin was in an amorphous form and that morphology changes occurred in comparison with pure curcumin and with the control. Whereas curcumin showed 10–60 mm cubic crystals, in drug/CaCO_3_ solid dispersion, cuboid crystals size decreased proving a better dispersion and the formation of small microspheres could be detected.

**Figure 3:**
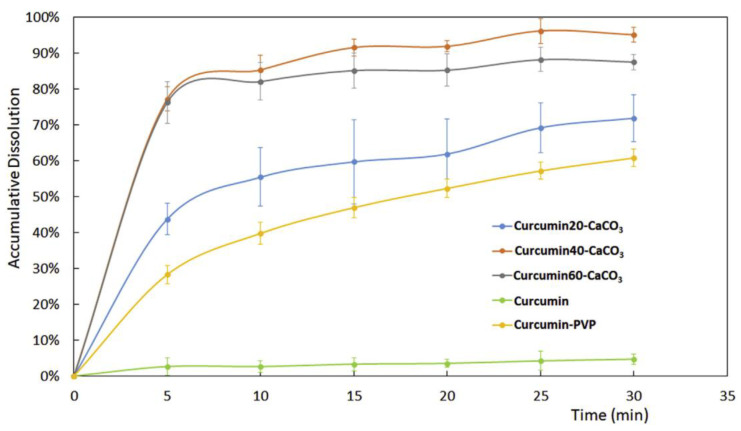
Dissolution profiles of curcumin from pure curcumin, curcumin-CaCO_3_ samples with increasing drug loadings and curcumin-PVP solid dispersion. Reprinted with permission from Ref. [29] 2019, Elsevier.

The in vitro dissolution of curcumin in pH 1.2 fluid showed that the curcumin release was significantly improved compared with that of curcumin. Better results in curcumin release were obtained for the sample containing about 30% *w*/*w* of curcumin. In this case, the drug release was almost complete after 25 min and was better than that obtained from a solid dispersion of curcumin/polyvinylpyrrolidone (PVP) with the same weight ratio of drug and carrier (Figure 3).

The stability of the sample was detected by measuring the antioxidant activity of curcumin after 6 months in storage and the results proved no antioxidant activity reduction in comparison to that of the as prepared curcumin/CaCO_3_ sample. This result suggests that CaCO_3_ could be a potential carrier also for shelf-life protection [29].

### 2.3. Solvent Evaporation Procedure

Praziquantel-calcite solid dispersions have been deeply investigated over concerns of polymorphic drug changes and the possible interactions between drug and carbonate [30], drug solubility and dissolution rates [31] and the optimization of the properties of CaCO_3_ [32]. Borrego-Sánchez et al. prepared a solid dispersion of praziquantel and CaCO_3_ in a calcite form (weight ratio 1/5 drug/calcite) by the solvent evaporation procedure in ethanol [30]. From chemical–physical characterizations, the formation of different drug polymorphs was observed, maybe due to the interactions between CaCO_3_ and the praziquantel carbonyl group. The physical mixture of the drug and CaCO_3_ did not show any chemical–physical change in comparison to the pure drug, confirming that the procedure used to obtain the solid dispersion affects the formation of interactions between the drug and CaCO_3_.

Successively, the solubility and dissolution improvement of praziquantel were investigated both in the acidic medium of HCl 0.001 M and in simulated intestinal fluid pH 6.8 [31]. The solubility tests showed an improvement of praziquantel solubility (1.42 mg/mL praziquantel solubility from solid dispersion vs. 0.45 mg/mL solubility of pure praziquantel). A dissolution test in acid medium showed an increase in the dissolution rate in agreement with what was observed in the solubility test. The drug release was 80% at 10 min and was complete within 1 h. This can be explained both with the rapid dissolution of CaCO_3_ in acid conditions and the changes in the praziquantel crystalline form. In vitro cytotoxicity of the composite was investigated towards the tumor cell line obtained from a colorectal carcinoma (HCT116) and the results showed that, at the tested concentrations (range between 6.4 nM–100 mM), the solid dispersion resulted as biocompatible [32].

### 2.4. Antisolvent Procedure

The effects of two different preparation procedures on a drug physical state, release and solubility were investigated by Donnadio et al. who prepared carbamazepine/CaCO_3_ composites with different drug loadings by antisolvent precipitation and ball mill [34]. The anti-solvent method consisted of inducing simultaneously the precipitation of the drug and CaCO_3_ by the fast addition of an aqueous Na_2_CO_3_ solution to an ethanolic solution of carbamazepine and CaCl_2_, with successive further dilution with water to reduce the mixture ethanolic grade and to induce complete drug precipitation. For comparison, samples of pure carbamazepine were prepared by the same procedures. From chemical–physical characterization, it emerged that the used procedures affected the drug polymorphic form. In fact, polymorph III was present in all samples obtained by ball mill, whereas in the case of antisolvent procedure, an unknown form was obtained at low drug concentrations (20 and 40% *w*/*w*) and polymorph IV was detected in the composite with the higher drug loading (70% *w*/*w*). Attention was drawn to the two samples containing about 40% *w*/*w* of drug loading as this amount is acceptable and allows the obtaining of formulations with a good drug dose. Composites obtained by the antisolvent method were very thin and small prisms deposited on the bigger cubic CaCO_3_ crystals. By ball milling, aggregates of carbamazepine particles recovered CaCO_3_ crystals almost completely. Thus, the shape of the drug crystals was affected more by the procedure than by the presence of CaCO_3_. From granulometric analysis it could be observed that the presence of CaCO_3_ decreases the powder size, and the frequency particle size range was narrower for the composites than for pure carbamazepine. Finally, composites prepared by the antisolvent method showed a very low crystallinity grade. Solubility tests, performed in HCl aqueous solution pH 1.2 at 37 °C for 2 h, showed the formation of a supersaturated solution with a supersaturation grade of 1.84 and 1.67 for the antisolvent solvent and ball milled sample, respectively.

**Figure 4:**
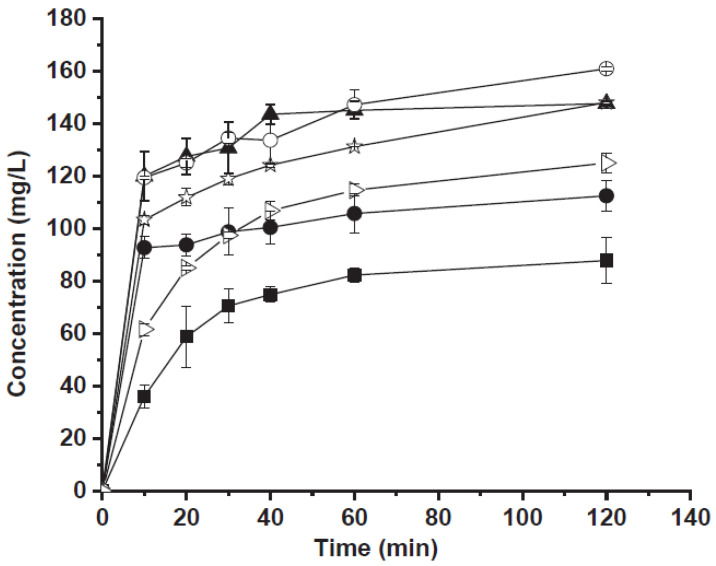
Carbamazepine release as a function of time from: commercial carbamazepine (full square), carbamazepine treated by ball mill (full circle), carbamazepine crystallized by antisolvent method (full triangle), physical mixture (empty triangle), carbamazepine/CaCO_3_ by ball mill (empty circle) and carbamazepine/CaCO_3_ by antisolvent procedure (empty stars). Reprinted with permission from Ref. [34] 2020, Elsevier.

The antisolvent composite showed the best in vitro release profile (Figure 4). The percentage of the released drug was about 76% after 30 min and was complete after 120 min. Probably, in the case of the ball milling sample, the presence of carbamazepine particle aggregates which almost completely recovered the surface of CaCO_3_ crystals, reduced the contact between the carbonate and the acidic medium, decreasing the carbonate dissolution rate. In the case of the antisolvent sample, the contact with acidic medium causes the rapid dissolution of CaCO_3_ allowing the release of small and not aggregated carbamazepine crystals. In conclusion the improved release of carbamazepine could be due to many factors such as (i) the smaller crystal size, (ii) changes in polymorphic form and (iii) reduced crystallinity. Moreover, following to CaCO_3_ solubilization in an acid environment, small drug crystals are released.

The antisolvent composite showed also other physical properties suitable for drug formulation, such as a narrow particle size range, that prevent powder mixture segregation ensuring drug content uniformity and excellent flowability (Carr’s index of 5.5%) [34].

## 3. Porous CaCO_3_

Amorphous solids, because of the molecules’ disordered arrangement, have an enhanced apparent solubility and dissolution rate in comparison with the crystalline form. Unfortunately, their use is limited by their instability. In fact, due to the high energy state of the amorphous form, it has a strong tendency to convert to the crystalline state, with a loss of the acquired advantages [40]. Among the strategies proposed for stabilizing the amorphous solids, the use of mesoporous compounds, such as ordered mesoporous silica, has been largely proposed. In these compounds, the pore diameter is so small that drug recrystallization is prevented, and this guarantees the drug physical stability [41,42]. Forsgren et al. investigated mesoporous CaCO_3_ nanoparticles as a stabilizer of amorphous celecoxib [35]. Mesoporous CaCO_3_ (mainly in vaterite polyphorm) particles loaded with celecoxib were prepared in one step by adding the drug directly in a methanolic solution during the CaCO_3_ synthesis. Samples with increasing celecoxib loadings were prepared. The presence of celecoxib in an amorphous form, with small traces of a crystalline form in the sample with the highest drug load, was detected. To evaluate the physical stability of the amorphous form, the composites were stored in two different conditions: (i) at 5 °C and dry conditions for 4 weeks and (ii) at 100 RH at room temperature for 7 days. Whereas the sample stored in dry and cool conditions did not undergo drug recrystallization, in the other samples, submitted to accelerated stress conditions, a partial celecoxib recrystallization occurred especially when the highest drug amount was present. This was explained by the transformation of the metastable vaterite into the more stable not porous calcite resulting in the expulsion of the drug from the pores and consequent recrystallization. The solubility test showed that celecoxib from samples with 8% and 15% *w*/*w* loading reached concentration 5 times higher than that obtained from crystalline celecoxib and from the sample that had a higher drug loading and containing celecoxib in crystalline form. This higher apparent solubility was maintained for 25 min and successively decreased. This research showed that mesoporous CaCO_3_ can prevent celecoxib recrystallization in a lack of humidity where the conversion of vaterite into calcite is slower.

As the specific surface area of a carrier is a key parameter for drug adsorption and its successive release, researchers directed their research to fine-tune the synthesis of calcium carbonates with a high specific surface area. Sun et al. synthesized a mesoporous CaCO_3_ with a high specific surface area by a stable and highly reproducible method which does not require the use of surfactants or additives [36]. This calcium carbonate was characterized by a high specific surface area of about 350 m^2^/g, and a pore volume of 0.86 cm^3^/g and 7.3 nm diameter. The poorly soluble drugs celecoxib and itraconazole were loaded using a solvent evaporation procedure. Drug loading was 14.49% *w*/*w* for celecoxib and 25.78% *w*/*w* for itraconazole. The loaded drug was in an amorphous form and was inside the pores with a partial decrease in porosity, but not a complete pore plugging. Itraconazole in vitro release in the simulated gastric fluid without enzymes (pH 1.2) was rapid and almost all the drug was released within 50 min., whereas the drug dissolution from crystalline itraconazole was very slow and after 6 h only 20 % of the drug had dissolved. Celecoxib release was evaluated at pH 6.8 in the phosphate buffer. Rapid drug release was observed and after 90 min. over 90% of the loaded celecoxib was released vs. a 13% from the crystalline drug. A negative feature of this porous CaCO_3_ was its low physical stability.

## 4. Porous Functionalized CaCO_3_ (FCC)

**Figure 5:**
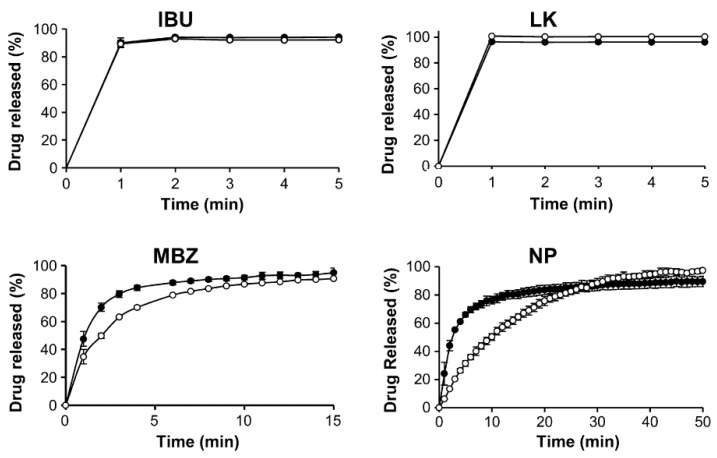
Drug release from drug loaded FCC (full circle) in comparison with FCC/drug physical mixture (empty circle). IBU = ibuprofen, LK = potassium salt of losartan, MBZ = metronidazole, NP = nifedipine. Reprinted with permission from Ref. [39] 2014, Elsevier.

Meanwhile, some researchers pointed their attention to porous functionalized CaCO_3_, which is a calcium carbonate functionalized by calcium phosphate and has been proposed as a new excipient. This material has been introduced as a novel excipient for orally dispersible tablets [43] and is characterized by high porosity, an enlarged surface area and it is able to adsorb a higher amount of fluid with a faster rate than traditional CaCO_3_ [44]. All these properties could provide high drug loading. Under the name of functionalized CaCO_3_, a series of co-precipitates from CaCO_3_ and calcium phosphate are included. They are of different types depending on the calcium phosphate content which ranges from 13 to 85% *w*/*w* and a porosity of approximately 60% (*v*/*v*) [45]. FCC particles consist of denser cores with smaller pores (core voids < 0.5 μm) surrounded by a network layer which possesses larger pores and channels (>0.5 mm). This gives meso- and macroporous material properties [46]. These excipients have been proposed not only for orally dispersible tablets but also for floating tablets [47], mucoadhesive delivery systems for colon targeting [48] and the delivery of proteins [49]. Thus, Preisig et al. investigated the possibility of using porous-functionalized CaCO_3_ as a drug carrier for drugs with different biopharmaceutical properties and chose as four model drugs belonging to three classes of BCS: ibuprofen and nifedipine (NP), which belong to class 2 of BCS and are characterized by poor water solubility and good permeability; potassium salt of losartan belonging to class III (good solubility and low permeability) and the prodrug metronidazole benzoate of class IV (poor solubility and poor permeability) [37]. As a functionalized CaCO_3_, among those which are disposable, an FCC named FCC S01 was chosen because of its large average particle size (17.9 mm) and highest porosity (70%). This matrix has both mesoporous and macroporous characteristics and its outer surface is characterized by interconnected “rose-like” petals that are larger than the pores present in the inner core. Thus, in the complex, it possesses a wide range of pore sizes ranging from 0.01 to 1 mm [37]. Drugs were loaded by the solvent evaporation method after drug solubilization in an organic solvent according to their solubility (Table 1). First of all, the best drug loading was chosen and for this purpose samples with increasing drug loadings (25–50% *w*/*w*) were prepared and those characterized by a lack of drug agglomerates were chosen. Good samples were obtained with a drug load of 40% *w*/*w* for metronidazole and ibuprofen and 35% *w*/*w* for nifedipine. The in vitro drug release test, performed in phosphate buffer pH 6.8 with the addition of sodium lauryl sulfate to maintain sink conditions, showed that faster drug release was obtained for nifedipine and metronidazole benzoate in comparison with the respective physical mixture and that the drug release was complete in half the time (Figure 5). This result was attributed mainly to the higher surface area of the samples.

Jonhson et al. investigated the effects of two other FCCs, namely FCC S07 and FCC S10, on the dissolution rate of the poorly soluble compounds curcumin and L-carvone with an application in pharmaceutics and nutraceuticals [38]. These FCCs have similar particle diameters and surface chemistry, but different pore volumes and pore sizes. In particular, FCC S07 is characterized by a specific surface area of 154.1 m^2^/g, a larger intraparticle pore volume (1.5 cm^3^/g) and a higher volume of very small pores (about 10 nm). FCC S10 had a specific surface area twice lower (77 m^2^/g) and an intraparticle pore volume of 1.1 cm^3^/g. The loading was performed by the incipient wetness procedure, by dripping the acetone curcumin solution or the L-carvone oil (without other solvent) into the FCC under mixing and repeating the operation several times until the loaded FCC powder maintained the same flowability and was not stick compared with pure FCC.

The L-carvone maximum load was 35% for FCC S07 and 38% for FCC S10. The release was performed in a phosphate buffer pH 6.8 with the adding of sodium laurylsulfate (1%) and official dissolution test apparatus was not used. L-carvone released from both loaded FCCs had almost the same profile and was very fast and complete after a few minutes. The release from the pure L-carvone was slower. This faster release may be due to the high interfacial area of the L-carvone inside or between FCC particles.

As concerns curcumin, its maximum loading was 15% *w*/*w*. All samples showed the lack of crystalline curcumin and showed a fast curcumin release (almost all curcumin was released within a few minutes) whereas complete curcumin release from the pure drug occurred after about 16 h.

**Figure 6:**
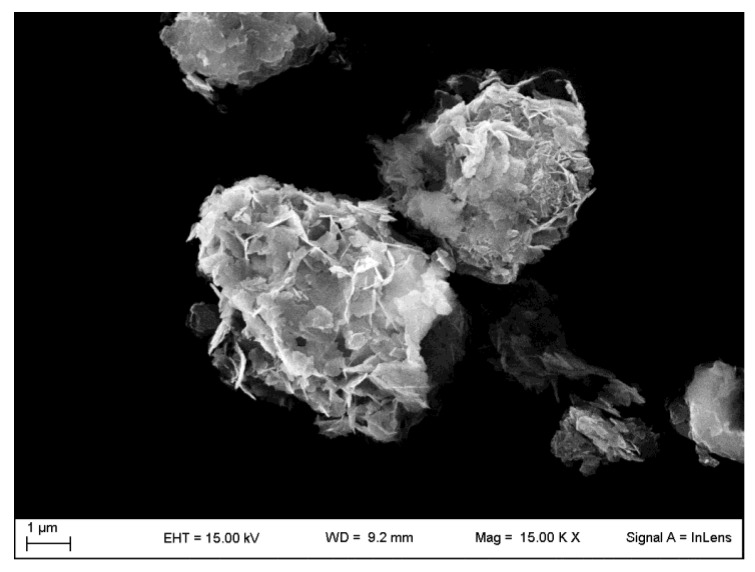
FE-SEM image of the FFC named Omyapharm 500 OG Omya, taken through a LEO 1525 ZEISS instrument (Jena, Germany).

Another method of drug loading has been investigated by Liu et al. [39]. They studied the physical state of the poorly water-soluble drug carvedilol loaded by ball milling mechanochemical activation. As with FCC, Omyapharm 500 OG was used [50]. A Field Emission Scanning Electron Microscopy (FE-SEM) image of Omyapharm 500 OG is reported in Figure 6. Samples with different weight ratios (10–90% *w*/*w* of drug) and milling times (maximum 90 min) were prepared. The pure drug became amorphous after 20 min milling, the carvedilol in 50% *w*/*w* drug-loaded sample became amorphous within 10 min, whereas FCC maintained its crystalline form.

From the solid state characterization of samples obtained from 90 min ball milling, it was highlighted that carvedilol was in a pure amorphous phase. When the loading was 30% *w*/*w* and below, the drug molecules were stabilized by the carbonate and recrystallization did not occur.

The physical stability test, performed in a dessicator for 19 weeks at 25 °C, proved the lack of recrystallization for a sample containing 10–30% *w*/*w* of carvedilol. An in vitro drug release at pH 6.8 in non-sink conditions was performed for crystalline and amorphous carvedilol and all the samples at different drug loadings. Drugs released from amorphous carvedilol showed at the first minute a higher drug concentration in comparison with the crystalline form. This initial improvement successively disappeared, maybe due to the conversion of the amorphous to the crystalline form. The highest supersaturation grade (3) was obtained with the carvedilol-CaCO_3_ samples with a drug load of 30 and 40% *w*/*w*. This supersaturation state was maintained for a long time, meaning that the presence of FCC improved the amorphous drug dissolution. From this investigation, it can be concluded that the ball milling procedure induces the transformation of crystalline drugs in an amorphous form and stabilized it for a long time, especially when the drug load is 30% *w*/*w*. This sample showed the best results also as concern drug dissolution which was fast and allowed to reach the highest drug concentrations for a long time [39].

## 5. Conclusions

Calcium carbonate could represent a good tool for improving poorly soluble drug dissolution rates. Its main advantages are (i) its safety, proved by its traditional use as an excipient; (ii) its low cost; (iii) its eco-friendly nature; (iv) the existence of different polymorphic states such as porous vaterite and (iv) the possibility of having porous forms with a high surface area. Considering these favorable characteristics, this excipient deserves to be investigated as an excipient for improving dissolution of BCS class two and four drugs, because it could be a valid alternative to other more expensive and less known new excipients. Drug/CaCO_3_ compositions are characterized by drug crystals with different morphologies in comparison with that of the pure drug, smaller sizes, and presence of the drug in amorphous or polymorphic forms. At the basis of these physical transformations, there are possible interactions among the drug molecules and the salt that have been proposed in some of the above-described investigations. It has to be highlighted that all these physical modifications are good prerequisites for obtaining an improvement in the drug dissolution rate. Moreover, following to the CaCO_3_ dissolution in an acidic medium, small drug crystals are rapidly released. The good effects on in vitro drug dissolution rate and the improvement of the apparent solubility are confirmed by the investigations here described. As amorphous and polymorphic forms have physical instability, studies aimed at verifying the stability of drug/calcium carbonate compositions should be carried out. Among the proposed procedures, higher drug loading can be obtained by ball mill, solvent evaporation and an antisolvent procedure. All these procedures lead to changes in the physical state of the drug, but it would be necessary to further investigate which of them favors the formation of mixtures with suitable drug-calcium carbonate interactions so as to prevent recrystallization, but to ensure a rapid release as the carbonate solubilizes in the gastric environment. The use of porous vaterite could offer further advantages such as the loading of high drug amounts, to maintain the drug dispersed in a high surface area and to increase the physical stability of amorphous forms when confined in a narrow space. Unfortunately, these advantages are hindered by the physical instability of this polymorph. However, due to the great industrial and biomedical interest in this polymorph, many researchers have turned their studies toward methods to improve vaterite stability [51]. Other attempts have been undertaken with the use of functionalized CaCO_3_ which joins porosity to good stability and promising results have been reported.

The papers published to date are characterized by different loading procedures and conditions and also in vitro dissolution tests are not performed in the same conditions (for example, sink or non-sink conditions, presence of surfactant, different dissolution fluids) and thus, notwithstanding the good results obtained concerning the apparent solubility and dissolution rates, no clear relationships can be drawn between the main characteristics of the material/loading procedure and dissolution behavior. Further studies are required to strongly support the findings described here and to evaluate the in vivo behavior.

## Figures and Tables

**Table 1:** Methods for obtaining carbonate-drug composites and main characteristics.

Drug	CaCO_3_ Form	Procedure	Drug Loading (% *w*/*w* ^a^ Unless Differently Indicated)	Drug/CaCO_3_ Physical Solid State	Ref. ^b^
Sulfathiazole	calcite	Ball milling	72 (2:1 M/M)	Mechano-composite sulfathiazole-CaCO_3_ (no physico- chemical characterization)	[27]
Naproxen	calcite and vaterite forms	Co-precipitation ^c^	3.4	-	[28]
Curcumin	calcite	Co-precipitation	15.8–42.6	Amorphous form At higher drug loadings small cuboid crystals	[29]
Praziquantel	calcite	Solvent evaporation	16.67	Polymorphic forms	[30,31,32]
Silybin	NanoCaCO_3_^d^ (calcite)	(1) Preparation of CaCO_3_ nanoparticles templated by polymeric micelles (2) Silybin adsorption	50	-	[33]
Carbamazepine	Calcite	Antisolvent (Ethanol/water) Ball milling	10–70	polymorphic form polymorphic form	[34]
Celecoxib	Vaterite mesoporous CaCO_3_ amorphous mesoporous CaCO_3_	One pot with CaCO_3_ preparation (Methanol) Solvent evaporation (Ethanol)	48–25 (*V*/*V*) 14.49	Amorphous form Amorphous form	[35] [36]
Itraconazole	Amorphous mesoporous	Solvent evaporation Dichloromethane	25.58	Amorphous form	[36]
Metronidazole benzoate	Porous FCC ^e^	Solvent evaporation (Acetone)	25–50	Amorphous form	[37]
Ibuprofen	Porous FCC ^e^	Solvent evaporation (Acetone)	25–50	Amorphous form	[37]
Losartan potassium ^f^	Porous FCC	Solvent evaporation (Methanol)	25–50	Amorphous form	[37]
Nifedipine	Porous FCC ^e^	Solvent evaporation (Acetone)	25–50	Amorphous form	[37]
L-carvone	Porous FCC ^e^	Incipient wetness (L-carvone oil without solvent)	1.3–35	-	[38]
Vanillin ^f^	Porous FCC ^e^	Incipient wetness (Acetone) Melting	1.3–35	Amorphous/crystalline depending on the drug loading and FCCs	[38]
Curcumin	Porous FCC ^e^	Incipient wetness (Acetone) Melting	1.3–35	Amorphous form	[38]
Carvedilol	Porous FCC ^e^	Ball milling	10–90	Amorphous form	[39]

^a^ Drug grams in 100 g of sample. ^b^ Ref.: reference. ^c^ Three different procedures based on co-precipitation were used, for details see the text. ^d^ nanoCaCO_3_ = CaCO_3_ nanoparticles. ^e^ FCC = functionalized CaCO_3_. ^f^ water soluble compound.

## Data Availability

Not applicable.
